# A novel three-dimensional template combined with MR-guided ^125^I brachytherapy for recurrent glioblastoma

**DOI:** 10.1186/s13014-020-01586-4

**Published:** 2020-06-08

**Authors:** Xiangmeng He, Ming Liu, Menglong Zhang, Roberto Blanco Sequeiros, Yujun Xu, Ligang Wang, Chao Liu, Qingwen Wang, Kai Zhang, Chengli Li

**Affiliations:** 1 Department of Interventional MRI, Shandong Medical Imaging Research Institute, Cheeloo College of Medicine, Shandong University, Shandong Key Laboratory of Advanced Medical Imaging Technology and Application, Jinan, Shandong People’s Republic of China; 2 The South Western Finland Imaging Centre, Turku University Hospital, Turku, Finland; 3 Department of Medical Imaging and Interventional Radiology, Affiliated Yantai Yuhuangding Hospital of Qingdao University, Yantai, Shandong People’s Republic of China; 4 Department of Tumor Minimally Invasive, Tai’an Central Hospital, Tai’an, Shandong People’s Republic of China; 5 Department of Ultrasound, Shandong Provincial Hospital Affiliated to Shandong University, Jinan, Shandong People’s Republic of China

**Keywords:** Printing, three-dimensional, Glioblastoma, Brachytherapy, Magnetic resonance imaging, Technology

## Abstract

**Background:**

At present, the treatment of recurrent glioblastoma is extremely challenging. In this study, we used a novel three-dimensional non-coplanar template (3DNPT) combined with open MR to guide ^125^I seed implantation for recurrent glioblastoma. The aim of this study was to evaluate the feasibility, accuracy, and effectiveness of this technique.

**Methods:**

Twenty-four patients of recurrent glioblastoma underwent 3DNPT with open MR-guided ^125^I brachytherapy from August 2017 to January 2019. Preoperative treatment plan and 3DNPT were made according to enhanced isovoxel T1-weighted MR images. ^125^I seeds were implanted using 3DNPT and 1.0-T open MR imaging guidance. Dosimetry verification was performed after brachytherapy based on postoperative CT/MR fusion images. Preoperative and postoperative dosimetry parameters of D90, V100, V200, conformity index (CI), external index (EI) were compared. The objective response rate (ORR) at 6 months and 1-year survival rate were calculated. Median overall survival (OS) measured from the date of brachytherapy was estimated by Kaplan-Meier method.

**Results:**

There were no significant differences between preoperative and postoperative dosimetry parameters of D90, V100, V200, CI, EI (*P* > 0.05). The ORR at 6 months was 75.0%. The 1-year survival rate was 58.3%. Median OS was 12.9 months. One case of small amount of epidural hemorrhage occurred during the procedure. There were 3 cases of symptomatic brain edema after brachytherapy treatment, including grade three toxicity in 1 case and grade two toxicity in 2 cases. The three patients were treated with corticosteroid for 2 to 4 weeks. The clinical symptoms related to brain edema were significantly alleviated thereafter.

**Conclusions:**

3DNPT combined with open MR-guided ^125^I brachytherapy for circumscribed recurrent glioblastoma is feasible, effective, and with low risk of complications. Postoperative dosimetry matched the preoperative treatment plan. The described method can be used as a novel implantation technique for ^125^I brachytherapy in the treatment of recurrent gliomas.

**Trial registration:**

The study was approved by the Institutional Review Board of Shandong Provincial Hospital Affiliated to Shandong University (NSFC:NO.2017–058), registered 1st July 2017.

## Introduction

At present, even after operation and radiochemotherapy, most malignant glioma patients eventually develop local or locoregional recurrence [1]. Median survival of patients with recurrent glioblastoma ranges from 3 to 6 months [2]. The treatment of recurrent glioblastoma is extremely challenging.

^125^I brachytherapy has been utilized as a salvage strategy for recurrent gliomas. The safety and efficacy of ^125^I brachytherapy as a local treatment option has been shown in recurrent gliomas [3–8]. Many studies have reported that patients with recurrent glioblastoma can benefit from ^125^I brachytherapy treatment with low rate of complications [3–5]. Accurate implantation of ^125^I seeds is one of the keys to the successful treatment of gliomas with ^125^I brachytherapy. It is believed that variances from the preoperative brachytherapy treatment planning may cause considerable variations in the radiotherapy outcome regarding tumor volume and normal tissue effects [9]. Stereotactic frame-guided ^125^I seed brachytherapy is the most widely used technique of this treatment. Despite the assistance of stereotaxy frame, the deviations in the space distribution of ^125^I seeds sometimes occur [10, 11]. On the other hand, due to the highly technical nature of the stereotactic implantation procedure, the clinical application of brachytherapy for gliomas is restricted to only a few experienced institutions. A new technology which is beneficial to optimize the brachytherapy procedure and reduce the technical difficulties is desired.

In recent years, a technique of three-dimentional non-coplanar printing template (3DNPT) has been applied to assist ^125^I seed implantation for tumors in head and neck, chest, abdomen, etc. It has been shown that the application of 3DNPT in brachytherapy can improve the accuracy and conformity of ^125^I seed implantation [12–15]. MR has excellent capabilities of soft tissue contrast, functional imaging and multi-planar imaging. It has significant advantages in target determination, intraoperative guidance and monitoring in craniocerebral surgery [16]. Therefore, in this study, we used a novel 3DNPT combined with open MR guidance in order to explore a new technique of ^125^I seed implantation for dose standardization, effectiveness, and a relatively easier procedure in the treatment of recurrent glioblastoma.

## Materials and methods

### Patients

Twenty-four patients of recurrent glioblastoma with 25 lesions received permanent ^125^I brachytherapy assisted by 3DNPT combined with 1.0-T open MR from August 2017 to January 2019 and were enrolled in this study, including ten females and fourteen males. Median age was 57 (37–73) years. All cases had undergone surgery or radiochemotherapy before the ^125^I brachytherapy treatment. In six cases the recurrent glioblastoma was confirmed by biopsy, and in eighteen cases by clinical follow-up and MRI. The median time to recurrence after initial radiochemotherapy was 6.8 months. The O^6^-methylguanine-DNA-methyltransferase (MGMT) promoter was methylated in 9 patients; the isocitrate dehydrogenase (IDH1) mutation was detected in 2 patients. The basic information of all the 24 cases is shown in Table 1.

**Table 1:** Characteristics of the 24 patients

Characteristic	Value
Sex (n)	
Male	14
Female	10
KPS^a^	60 (50–90)
Age (years)^a^	57 (37–73)
Tumor diameter (cm)^a^	3.7 (2.0–8.5)
Tumor location	
Frontal lobe	7
Parietal lobe	8
Occipital lobe	2
Temporal lobe	8
Previous treatment	
S + R + T	20
R + T	4
Prescribed dose (Gy)^a^	140 (120–160)
MGMT status	
Methylated	9
Unmethylated	8
Unknown	7
IDH1 mutation	
Mutated	2
Wild type	17
Unknown	5

*S* Surgery, *R* Radiotherapy, *T* Temozolomide; ^a^ = median (range)

Eligibility criteria included: (1) patients with glioblastoma who had previously undergone surgery and/or radiotherapy with temozolomide treatment; (2) recurrent supratentorial glioblastoma confirmed by pathology or MRI; (3) the number of tumor lesions ≤2; (4) life expectancy > 2 months. Exclusion criteria included: (1) diffusely infiltrative tumors, or tumors with infiltration of corpus callosum; (2) severe coagulation dysfunction (activated partial thromboplastin time of twice more than the normal time, or international standard ratio > 1.5, or platelet count < 50 × 10^9^/L); (3) unconscious patients; (4) patients with contraindications for MRI. This study was approved by the institutional review board, and written informed consent was obtained from all patients.

### Equipment and technique

^125^I seeds (Shanghai Xinke Pharmaceuticals, Shanghai, China) with half-life time of 59.4 days and initial dose-rate of 7.7 cGy/h were used in brachytherapy. A Brachytherapy Implantation Planning System (Beijing University of Aeronautics and Astronautics, Beijing, China) was utilized. ^125^I brachytherapy for recurrent glioblastoma was carried out under the guidance of a 1.0-T open MR scanner (Philips Healthcare, Amsterdam, Netherlands). A MR-compatible liquid crystal monitor (Philips Healthcare, Amsterdam, Netherlands) was used to display intraoperative images. A MR-compatible electrocardiogram monitor (Mammendorfer Institute of Physics and Medicine GmbH, Munich, Germany) was equipped to monitor vital signs of patients. A high-speed drill with the diameter of 1.9 mm was used to penetrate the skull. MR-compatible coaxial needles with a blunt tip (18-G, Wanlin Medical, Qingdao, China) were used for the implantation of ^125^I seeds. 3DNPT (Zhuoye Electronic Technology Co., Ltd., Zibo, China) with an accuracy of 0.1 mm was used to assist brachytherapy procedure.

### Preoperative preparation

Three fish oil capsule markers were placed and marked on the surface of the scalp as the alignment reference points of the template before preoperative imaging with MRI. Contrast-enhanced isovoxel T1-weighted images with slice thickness of 1 mm were obtained for preoperative planning. The preoperative brachytherapy treatment plan was performed subsequently. Gross Tumor Volume (GTV) was delineated according to the enhanced margin of tumor in each slice of the contrast-enhanced isovoxel T1-weighted images. According to previous literature, we determined clinical target volume (CTV) contour 10 mm out of the GTV [1, 3, 9]. Low activity ^125^I seeds of 0.8 mCi were utilized. The prescribed dose was 120–160 Gy. According to the size and shape of the target volume, the needle paths, the number of the seeds and their spacing were determined. Dose volume histogram and dosimetry parameters (D90, V100, V200, CI, EI) were obtained. The parameter of conformity index (CI) was used for evaluating the conformity of dose distribution. External index (EI) was used for evaluating the coverage of brain tissue outside CTV by prescribed dose. The equations of CI and EI referred to reported literature [13, 17, 18]. CI = (V_TP_ / V_T_) × (V_TP_ / V_P_), wherein, V_T_, V_TP_, and V_P_ were the target volume, the volume covered by the prescribed dose in the target volume, and the total volume covered by the prescribed dose, respectively. CI ranges from 0 to 1; with the increases of CI value, the dose conformity improves correspondingly. EI = (V_P_ –V_TP_) / V_T_ × 100%. EI ranges from 0 to 100%; with the decreases of EI value, the volume of normal brain tissue covered by prescribed dose decreases.

**Fig. 1:**
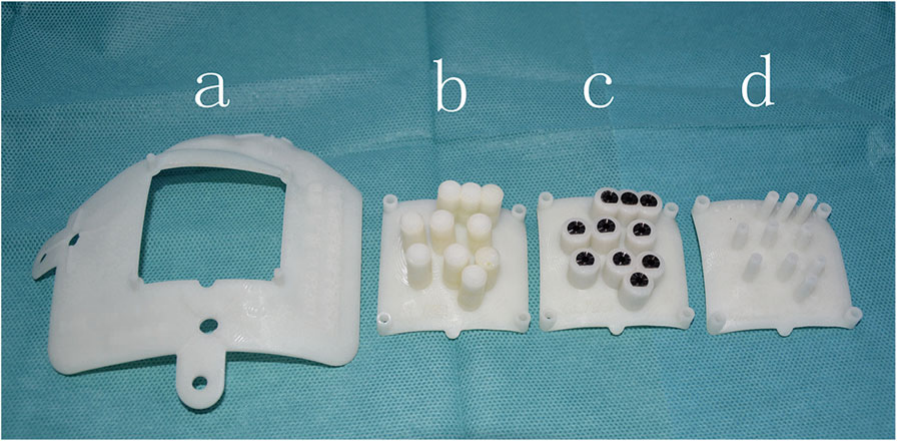
The structure of 3DNPT. **a** The base plate. **b** The positioning plate. **c** The drilling plate. **d** The puncturing plate. The three plates **(b, c, d)** are of the same size and can be embedded in and removed from the base plate

**Fig. 2:**
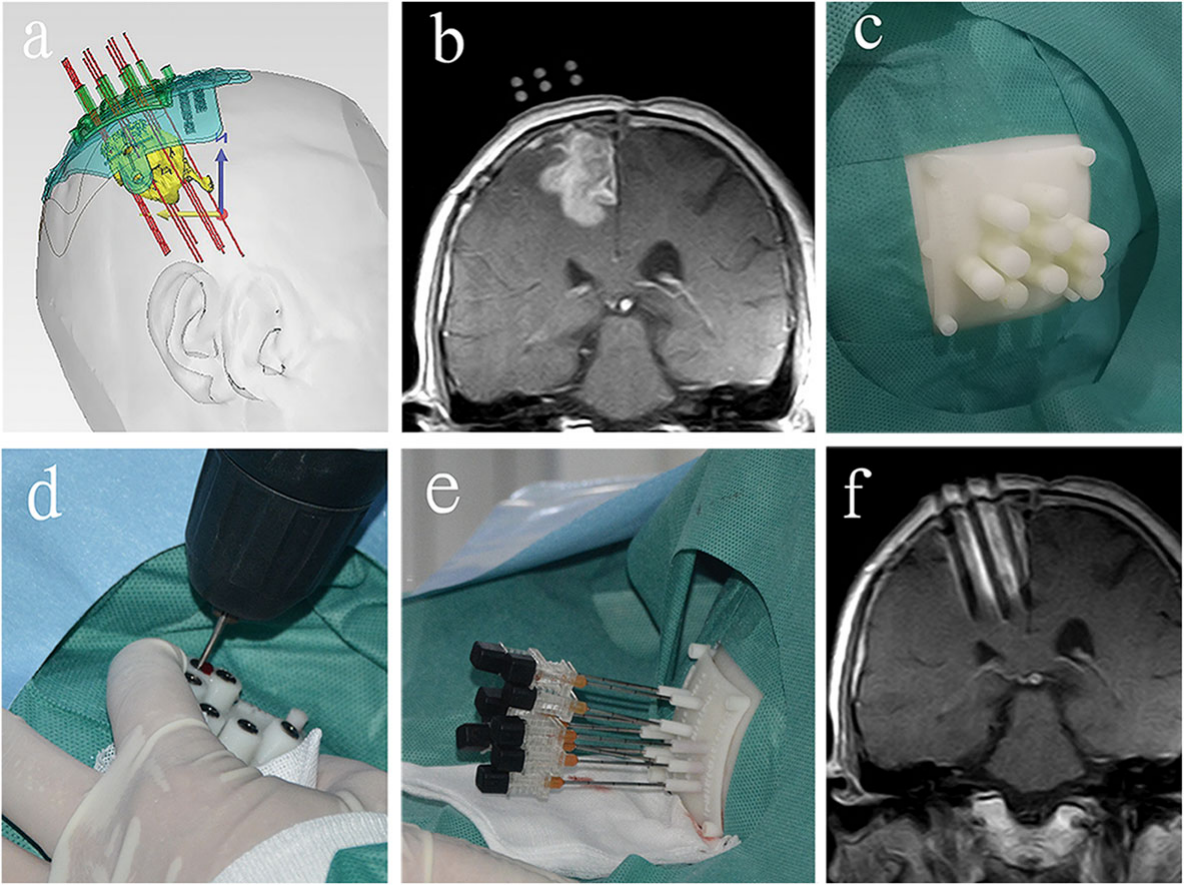
The procedure of 3DNPT combined with MR-guided ^125^I brachytherapy for recurrent glioblastoma. This patient had undergone surgery, radiotherapy, and temozolomide treatment before brachytherapy. **a** Preoperative design of 3DNPT. **b** The markers in the positioning template and the tumor were displayed in MRI. **c** The positioning template was placed and fixed on the scalp. **d** Twist-drill holes were made through drilling template. **e** Needles were punctured into the tumor through the puncturing template. **f** The position of needles could be displayed by the inoperative MRI

A 3DNPT was printed by a 3-D printer (Uniontech Co., Ltd) according to the preoperative brachytherapy treatment plan and contrast-enhanced isovoxel T1-weighted images (Fig. 1, Fig. 2a). The 3DNPT is comprised of a base plate and three movable plates that can be embedded in the base plate. The three movable templates include a positioning template, a drilling template, and a puncturing template (Fig. 1). The positioning template is filled with fish oil capsule markers, which display clearly in MRI. There is a sleeve for drilling made of titanium alloy and compatible with MR embedded in the drilling template. The puncturing template is used to guide the needles to puncture into the tumor through the twist-drill holes in the skull.

### Brachytherapy procedure

The positioning template was embedded in the base plate and placed on the scalp coinciding with the reference points. Then the patient underwent enhanced MR imaging. Enhanced images of T1-weighted turbo spin echo (T1W-TSE) were used to display the tumor and the markers in the positioning template (Fig. 2b). If the relative position of the template and the tumor was different from the preoperative plan, the position of template was fine-tuned. The template was then fixed by sterile patches (Fig. 2c) and the positioning template was removed from the base plate and replaced by drilling template. The patient was moved outside of the magnet bore out of 5 G area and given local anesthesia using lidocaine (2%) and intravenous conscious sedation using diazepam (5 mg). Twist-drill holes to the skull were made through drilling template using a battery-operated twist drill (Fig. 2d). Then the drilling template was replaced by the puncturing template. Needles with blunt tip were pushed into the tumor through the puncturing template (Fig. 2e). The patient was then moved back into the magnet bore for MR scanning to display the position of needles (Fig. 2f). Needle orientation was fine-tuned if necessary according to the MR images. The ^125^I seeds were then implanted into the tumor and with distribution following the preoperative brachytherapy treatment plan. After implantation of the ^125^I seeds, the patient underwent a final MR scanning of the entire brain to observe whether possible complications occurred.

### Postoperative dosimetry verification

**Fig. 3:**
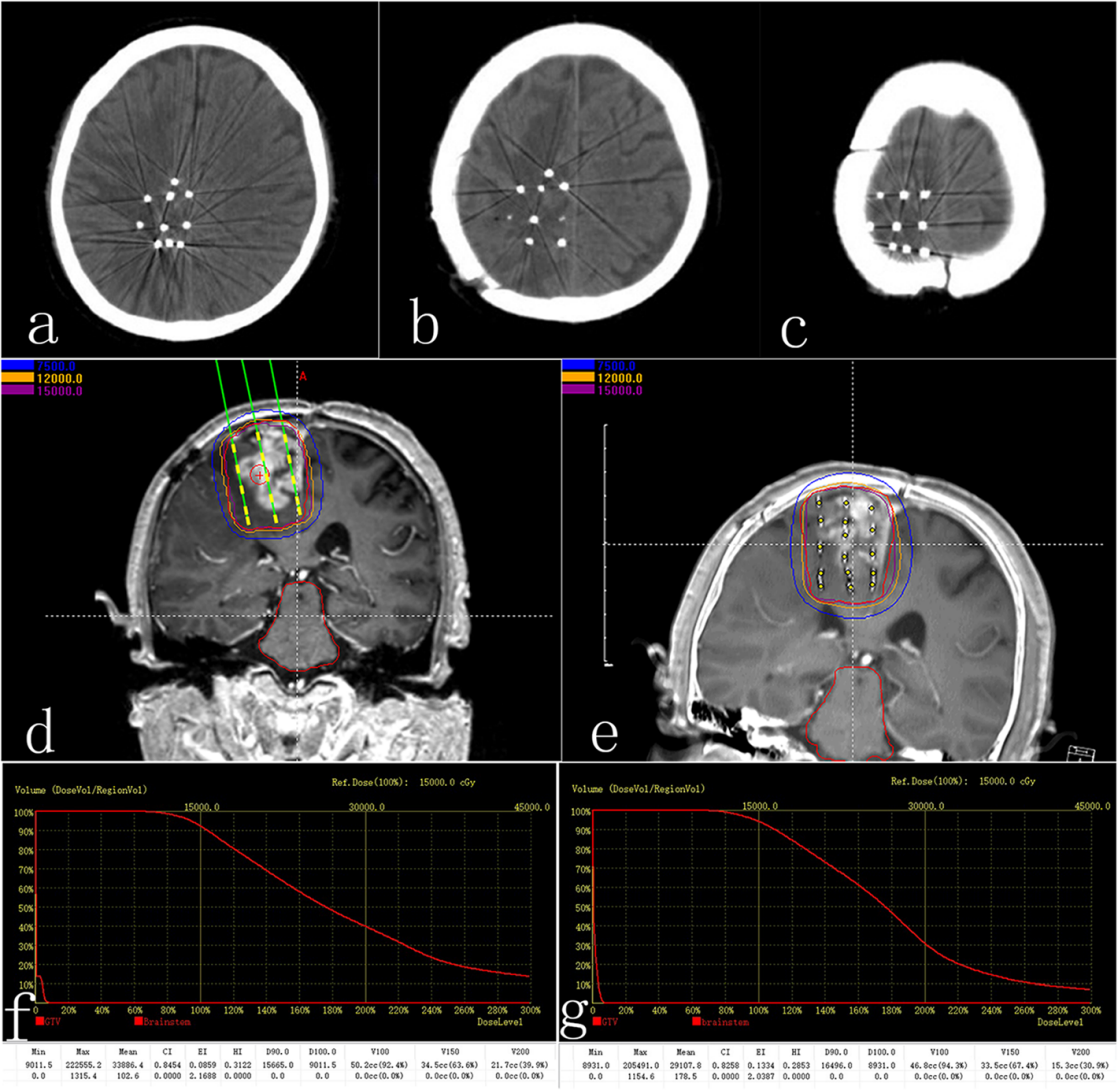
Preoperative plan and postoperative dosimetry verification. **a, b, c** Postoperative CT images showed ^125^I seed distribution**. d** Preoperative plan. **e** Postoperative dosimetry verification based on postoperative CT/MR fusion images. **f** Dose volume histogram of preoperative plan. **g** Postoperative dose volume histogram. Postoperative dosimetry matched the preoperative treatment plan

On the second day after the brachytherapy treatment, the patient underwent CT and MR scanning. CT and MR images were then fused and fusion images were transferred to brachytherapy treatment planning system for postoperative dosimetry verification (Fig. 3). Dose volume histogram and dosimetry parameters (D90, V100, V200, CI, EI) were obtained.

### Follow-up

Patients were followed up by MRI every 2 to 3 months. According to response assessment in neuro-oncology (RANO), the treatment response at 6 months was evaluated as complete response (CR), partial response (PR), stable disease (SD), and progressive disease (PD). Toxicities were evaluated with Radiation Therapy Oncology Group (RTOG)/European Organization for Research and Treatment of Cancer (EORTC) and Common Terminology Criteria for Adverse Events (CTCAE) v4.0.

### Statistical analysis

Preoperative and postoperative dosimetry parameters were compared by paired t-test or nonparametric Wilcoxon test. The overall response rate (ORR) at 6 months was calculated as CR + PR. The 1-year survival rate was calculated. Median overall survival (OS) measured from the date of brachytherapy was estimated by Kaplan-Meier method. The ORR and OS in MGMT methylated and unmethylated patients were compared by Fisher’s exact test and log-rank test. Statistical analysis was performed by SPSS 22.0 statistical software. *P* values < 0.05 was considered to indicate significant difference.

## Results

### Dosimetry verification

The preoperative D90, V100, V200, CI, and EI were 149.3 ± 13.6 Gy, 91.8 ± 1.2%, 43.9 ± 4.9%, 0.77 ± 0.04 and 18.3 ± 5.9% respectively. The postoperative D90, V100, V200, CI, and EI were 148.8 ± 14.3 Gy, 91.6 ± 1.3%, 44.8 ± 4.8%, 0.76 ± 0.04 and 19.7 ± 6.8% respectively. There were no significant differences between preoperative and postoperative dosimetry parameters of D90, V100, V200, CI, EI (*P* > 0.05) (Table 2).

**Table 2:** Comparison of preoperative and postoperative dosimetry parameters

Parameter	Preoperative			Postoperative			*P* value
	Range	Median	M (SD)	Range	Median	M (SD)	
D90 (Gy)	120.4–177.5	148.5	149.3 (13.6)	121.6–178.2	147.5	148.8 (14.3)	0.639
V100 (%)	90.0–94.7	91.7	91.8 (1.2)	90.1–95.0	91.3	91.6 (1.3)	0.488
V200 (%)	31.4–49.7	45.2	43.9 (4.9)	30.9–49.3	46.5	44.8 (4.8)	0.276
CI	0.69–0.85	0.77	0.77 (0.04)	0.67–0.83	0.76	0.76 (0.04)	0.067
EI (%)	8.6–29.3	18.5	18.3 (5.9)	10.9–36.6	18.7	19.7 (6.8)	0.076

*M* Mean, *SD* Standard deviation

### Local control and survival

**Fig. 4:**
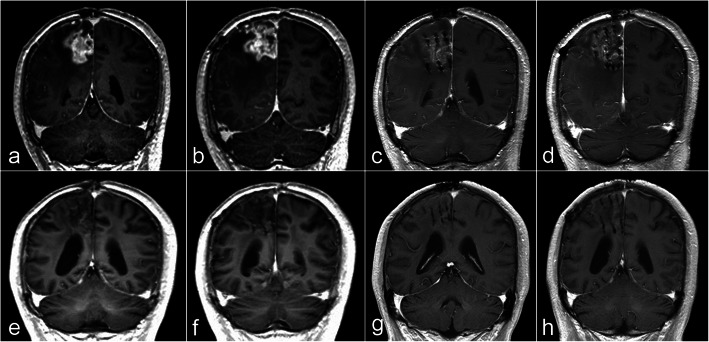
Postoperative follow-up of the case shown in Fig. 2 by MRI. **a, b** MR images before ^125^I brachytherapy treatment. **c, d** Enhanced T1W images 2 months after ^125^I brachytherapy shows that the enhancement of the tumor decreased significantly. **e, f** T1W images 6 months after brachytherapy shows that the enhancement of the tumor further decreased. The brain edema around the tumor was significantly decreased. **g, h** T1W images ten months after brachytherapy shows that there is no obvious enhancement of the tumor, which indicates that the tumor is well-controlled

**Fig. 5:**
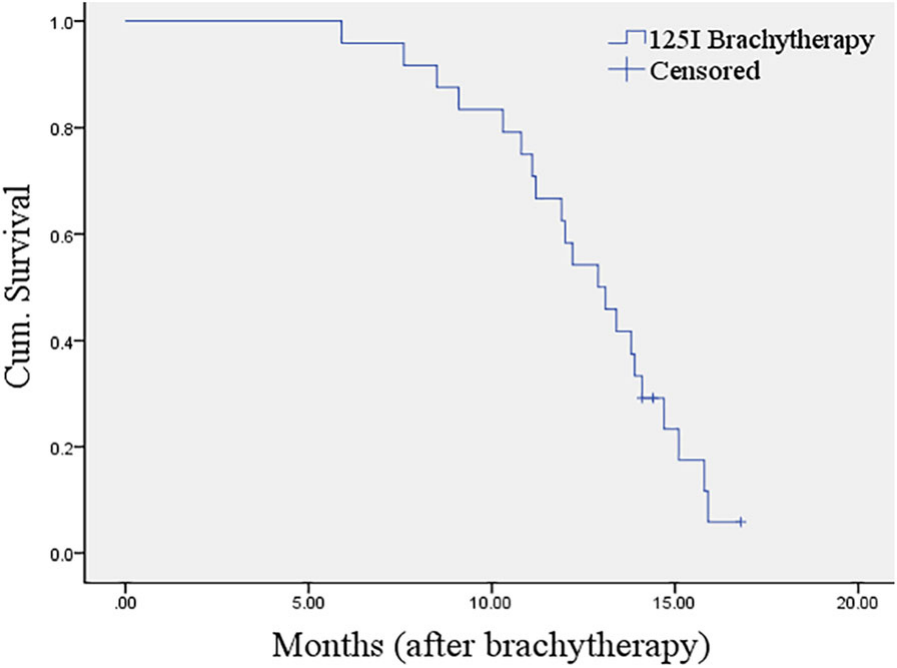
Kaplan-Meier plot for overall survival after ^125^I brachytherapy

The median follow-up period after brachytherapy was 13 months (range, 5.9–16.8 months). Three patients were alive at the point of this analysis. There was 1 case of CR, 17 cases of PR, 2 cases of SD, and 4 cases of PD at 6 months (Fig. 4). The ORR at 6 months was 75.0% (18/24). The 1-year survival rate was 58.3% (14/24). Median OS was 12.9 months (95% confidence interval, 11.2–14.6) (Fig. 5).

**Fig. 6:**
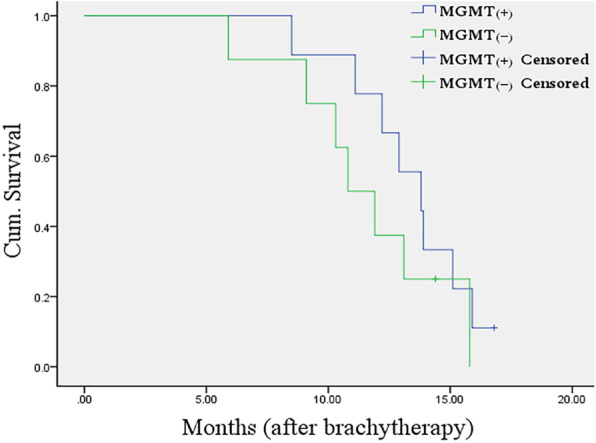
Kaplan-Meier plot for overall survival in MGMT methylated and unmethylated group after ^125^I brachytherapy

The ORR at 6 months in MGMT methylated and unmethylated patients were 77.8% (7/9) and 75.0% (6/8) respectively. There was no significant difference between the two groups (*P* = 1.000). The median OS in MGMT methylated and unmethylated patients were 13.8 months and 10.8 months respectively. There was no significant difference between the two groups (*P* = 0.286) (Fig. 6). The two patients with IDH1 mutation were PR at 6 months and the OS were 13.4 months and 14.1 months respectively.

### Complication and toxicity

There was 1 case of epidural hemorrhage with the amount of about 2.8 ml. No increase in bleeding was found by MRI 24 h after brachytherapy. No neurologic deficit and other complications occurred during the procedure and 1 day follow-up period. There were 3 cases of symptomatic brain edema requiring medication treatment within 2 weeks after brachytherapy treatment, including grade three toxicity in 1 case and grade two toxicity in 2 cases. The three patients were treated with corticosteroid for 2 to 4 weeks. The clinical symptoms related to brain edema were significantly alleviated thereafter. In addition to brain edema, there was 1 case of grade one pain of upper limb with no treatment.

## Discussion

Presently, stereotactic frame-guided ^125^I seed implantation is the most widely used technique of ^125^I brachytherapy for gliomas. Despite the assistance of stereotaxy frame, the deviations in the space distribution of ^125^I seeds are common [10, 11]. Viola A reported that the brain tumor volume covered by the prescribed dose after stereotactic ^125^I brachytherapy was significantly different from that of planned before implantation (75.8% vs 92.4%), and so was the conformity index (0.37 vs 0.54). The position of ^125^I seeds or catheters required adjustment in 20% of all the cases [10]. As accurate distribution of ^125^I seeds is the premise of therapeutic effect for gliomas, new techniques which help to optimize the ^125^I seed implantation procedure are of interest.

^125^I brachytherapy is widely used and has become one of the standard treatments for early prostate cancer. One of the reasons is that the application of planar template used in brachytherapy procedure helps the dose distribution in prostate cancer reach the expected standard [19–21]. In recent years, researchers have utilized 3DNPT to assist brachytherapy for other tumors, such as tumors of the head and neck, chest, abdomen, etc. It has been reported that with the assistance of 3DNPT, ^125^I seed implantation could achieve good accuracy as the template enables accurate alignment between the template and therapy area as well as accurate control of the implantation needles [12–14]. Presently, to the best of our knowledge, there has not been 3DNPT adaptation for brachytherapy of brain tumors. The skull provides an obstacle for brachytherapy. Because of the skull, the template needs to be facilitate drilling twist-drill holes on the skull. To this end we embedded a metal sleeve made of titanium alloy in the template. In order to display planned needle trajectory on 3DNPT on MR images, fish oil capsule markers were also put into the template. This made it possible to use 3DNPT combined with MR to guide the brachytherapy procedure.

The template provides for vital features, including needle track and alignment reference points. In our study, the needle track was accurately aligned with the target lesion through the alignment between the template and reference points on the scalp of therapeutic area. Needles could be accurately forwarded into the tumor. Dosimetry verification showed that there were no statistical differences between preoperative and postoperative dosimetry parameters of D90, V100, and V200. The results show that the planned dosimetry could be achieved ideally with the assistance of 3DNPT. CI was smaller, EI was larger than those in preoperative plan, but there were no statistical differences, which indicated that the conformity of preoperative planning could also be achieved by the use of 3DNPT. Compared to Viola et al. results, the dosimetry parameters of this study better achieved the preoperative plan, which shows that 3DNPT assisted ^125^I seed implantation is accurate.

In most of the reports on ^125^I seed implantation guided by stereotactic frame, a small number of catheters (1–4 catheters) or trajectories with implantation of relatively high activity ^125^I seeds (often > 3 mCi) were utilized (it is generally considered that high activity is > 20 mCi; the relatively high activity mentioned here is relative to the activity of < 1 mCi) [6, 22–24]. The use of relatively high-activity ^125^I seeds could reduce the number of seeds or trajectories needed for implantation, however, it be difficult to achieve sufficient conformity of dose distribution for irregular-shaped tumors. In dosimetric studies of ^125^I brachytherapy for prostate cancer it has been shown that using low activity ^125^I seeds (< 0.9 mCi) could achieve better conformity of dose distribution and reduce the dose received by normal tissue [25, 26]. In our study, we used low activity ^125^I seeds (0.8 mCi) for the treatment of recurrent glioblastoma. When implanting low activity ^125^I seeds into large tumors, more needle paths and twist-drill holes are necessary. In our case, due to the application of 3DNPT, drilling process could be accomplished at one time. This contributes to the rapidity of drilling process and the whole brachytherapy procedure is more straight forward. In contrast, the procedure of stereotactic ^125^I implantation requires adjusting coordinates of the frame repeatedly and drilling holes one by one in this case. Furthermore, stereotaxy frame needs to be installed before brachytherapy procedure. Compared with stereotactic ^125^I brachytherapy, 3DNPT-assisted brachytherapy reduces technical difficulties of implantation procedure. It is relatively easier to perform and is beneficial for clinical application of brachytherapy for recurrent gliomas.

At present, the treatment of template-assisted ^125^I seed implantation for tumors is typically carried out with CT guidance. However, due to the complexity of craniocerebral anatomy and the shape characteristics of skull, many brain tumors cannot be assessed via the transverse axis path, but should be approached using coronal or sagittal path for ^125^I seed implantation. At these planes the utility of CT guidance is limited, instead, MR has advantage in this aspect due to its multi-planar imaging capabilities. In addition, MRI benefits from its good soft tissue contrast with obvious advantages over CT in displaying brain lesions, and has been widely used in the guidance of brain biopsy and neurosurgery [27–30]. We used MR combined with 3DNPT to guide the ^125^I brachytherapy for recurrent glioblastoma. During the brachytherapy procedure, MR scanning was performed to confirm the position of puncture needles. If there was deviation of the needle or detection brain shift, fine-tuning of the needle was performed until the needle reached the correct position. The brachytherapy procedure was accomplished with the monitoring of MRI displaying the direction of the needles in two orthogonal MRI planes to ensure the accuracy of the direction of the needles. On the displaying of the ^125^I seeds, in MR images these are not as clearly visible as in CT images. To solve this problem, we used fusion of CT and MR images.

In this study, the ORR of 75% at 6 months indicated that favourable local control for recurrent glioblastoma could be achieved by the 3DNPT combined with MR-guided ^125^I brachytherapy. The OS was 12.9 months, which was better than what was reported in previous literature: the OS in literature on stereotactic ^125^I brachytherapy for recurrent glioblastoma was 7.6–10.8 months [4–6, 31, 32]. The result is considered to be related to the substantial increase in the prescribed dose we adopted. Previously reported prescribed dose of stereotactic or CT-guided ^125^I brachytherapy for recurrent glioblastoma was 50–110 Gy, while in our study, it was 120–160 Gy. Benefiting from the assistance of 3DNPT, better conformity of dose distribution was obtained, and less damage to normal brain tissue could be achieved. This was also one of reasons the increase of prescribed dose did not induce excessive toxicities in our study. Kickingereder assessed the benefits of stereotactic ^125^I brachytherapy with a prescribed dose of 60 Gy for 98 patients with recurrent glioblastoma. The median activity of ^125^I seeds was 16.1 mCi and the conformity index was 0.65. The median OS was 10.4 months [4]. Compared with Kickingereder’s research, the prescribed dose in our study was increased by 60 to 100 Gy, and the conformity index was increased by 0.11; the median OS was increased by 2.5 months and without toxicities that couldn’t be alleviated by medication treatment. Another aspect that the efficacy of ^125^I brachytherapy in this study can be attributed to is the exclusion of diffusely infiltrative recurrent glioblastoma, tumors with infiltration of the corpus callosum, and infratentorial tumors, which are considered to be unsuitable for localized treatment by ^125^I seed implantation [23, 33]. The patient pre-selection in this study indicated that the clinical effectiveness of this novel implantation technique is demonstrated in circumscribed recurrent supratentorial glioblastoma. Presently, the rate of reoperation of recurrent glioblastoma is below 30% according to reported literature [34]. For a larger proportion of recurrent glioblastomas that can not be resected totally or near totally, ^125^I seed implantation is a reasonable treatment due to its effectiveness and minimally invasive nature. The novel 3DNPT-assisted implantation technique could be considered as an alternative.

MGMT promoter methylation status and IDH mutations are important prognostic biomarkers for gliomas. A large number of reports have confirmed that MGMT methylation has impact on the therapeutic effect of the temozolomide chemotherapy and radiotherapy for high-grade gliomas [35–37]. In this study, we conducted a statistic analysis from the perspective of MGMT methylation status. The ORR at 6 months in MGMT methylated and unmethylated group were similar and did not show significant difference. This indicated that ^125^I brachytherapy could achieve relatively good local control for both MGMT methylated and unmethylated patients. The OS in the MGMT methylated group was much better than that in unmethylated group. Although there was no significant difference between the two groups, it seemed to be a trend of better survival in MGMT methylated group. It was possible that the statistic result related to the sampling error caused by the small sample size. The OS in the two patients with IDH1 mutation in this study were longer than the median OS of the whole cohort. It was consistent with the result that glioblastoma with IDH mutation have better prognosis reported in relevant literature [38, 39]. MGMT promoter methylation status and IDH mutation can be considered to be further studied as prognostic factors for recurrent glioblastoma patients who received ^125^I brachytherapy treatment.

In this study, there was 1 case of small amount of epidural hemorrhage, without large amount of hemorrhage and neurologic deficit, which suggested that the implantation procedure was safe. Blood vessels are readily displayed in the preoperative and intraoperative enhanced MRI, and can be taken into account and avoided in preoperative planning and brachytherapy process; in addition, the diameter of the drill used in this study is small (1.9 mm). These reasons undoubtedly contributed for the safety of this study. The main toxicity in this study was symptomatic grade two and grade three brain edema, which was significantly alleviated after corticosteroid therapy. Although all patients had undergone radiotherapy previously, ^125^I brachytherapy did not induce refractory brain edema or radiation necrosis, which is better than many previous reports. Gaspar performed stereotactic ^125^I brachytherapy for 59 patients with recurrent malignant gliomas (22 nonglioblastoma malignant gliomas, 37 glioblastoma). Eight point 5 % (5/59) of patients underwent reoperations for radiation necrosis and skull infection [6]. In our study, the low risk of complications can be attributed to three aspects. First, better conformity of dose distribution can be achieved by the assistance of 3DNPT, thus, dose received by normal brain tissue and damage to normal brain tissue can be reduced. Second, ^125^I seeds we used are of low dose-rate, so the normal brain tissue at the boundary of the treatment volume has greater opportunity to repair during the course of the treatment [40]. Third, the dose of ^125^I seeds decreases rapidly from the centre of the therapeutic volume towards the periphery.

There are limitations in our study. First, the number of cases is small. It needs to be increased in subsequent study. Second, this study is not a randomized controlled study. Randomized control design and comparison with other therapeutic methods can be used in further studies.

## Conclusions

In summary, this novel 3DNPT combined with MR-guided ^125^I brachytherapy for circumscribed recurrent glioblastoma is feasible, effective, and with low risk of complications. Postoperative dosimetry matched the preoperative treatment plan. The described method is relatively easier to perform. It can be used as a novel implantation technique for ^125^I brachytherapy in the treatment of recurrent gliomas.

## Data Availability

The datasets used and/or analyzed are available from the corresponding author on reasonable request.

## Abbreviations

3DNPT: Three-dimentional non-coplanar printing template
^125^I: Iodine-125
MGMT: O^6^-methylguanine-DNA-methyltransferase
IDH: Isocitrate dehydrogenase
KPS: Karnofsky performance status
GTV: Gross tumor volume
CTV: Clinical target volume
CI: Conformity index
EI: External index
T1W-TSE: T1-weighted turbo spin echo
OS: Overall survival
ORR: Objective response rate
RANO: Response assessment in neuro-oncology
CR: Complete response
PR: Partial response
SD: Stable disease
PD: Progressive disease
RTOG: Radiation Therapy Oncology Group
EORTC: European Organization for Research and Treatment of Cancer
CTCAE: Common Terminology Criteria for Adverse Events
